# DEFENDER: Detecting and Forecasting Epidemics Using Novel Data-Analytics for Enhanced Response

**DOI:** 10.1371/journal.pone.0155417

**Published:** 2016-05-18

**Authors:** Nicholas Thapen, Donal Simmie, Chris Hankin, Joseph Gillard

**Affiliations:** 1 Institute for Security Science and Technology, Imperial College London, London, United Kingdom; 2 Defence Science and Technology Laboratory, Porton Down, Salisbury, United Kingdom; University of Vermont, UNITED STATES

## Abstract

In recent years social and news media have increasingly been used to explain patterns in disease activity and progression. Social media data, principally from the Twitter network, has been shown to correlate well with official disease case counts. This fact has been exploited to provide advance warning of outbreak detection, forecasting of disease levels and the ability to predict the likelihood of individuals developing symptoms. In this paper we introduce DEFENDER, a software system that integrates data from social and news media and incorporates algorithms for outbreak detection, situational awareness and forecasting. As part of this system we have developed a technique for creating a location network for any country or region based purely on Twitter data. We also present a disease nowcasting (forecasting the current but still unknown level) approach which leverages counts from multiple symptoms, which was found to improve the nowcasting accuracy by 37 percent over a model that used only previous case data. Finally we attempt to forecast future levels of symptom activity based on observed user movement on Twitter, finding a moderate gain of 5 percent over a time series forecasting model.

## Introduction

The recent increases in global travel and the interconnected nature of modern life have led to an increased focus on the threat of diseases, both established and newly emerging. Public health officials need timely and accurate information on disease outbreaks in order to put measures in place to contain them. Traditional disease surveillance techniques such as reporting from clinicians take 1-2 weeks to collate and distribute, so finding more timely sources of information is a current priority.

In recent years social media, especially Twitter data, has been used to positive effect for: disease nowcasting (predicting the current level of illness from previous clinical data and current social data) [1–5], outbreak detection [6–9] and predicting the likelihood of individuals becoming ill [10]. News media has also been used to give early warning of increased disease activity before official sources have reported [11], as well as for giving an indication of the change in reproductive number of an outbreak [12].

This paper presents a software system, DEFENDER, which leverages social media to provide a combined disease outbreak detection and situational awareness capability. Much of the previous research in these areas focuses on specific conditions, with influenza being the most studied. Our approach seeks to generalise by focusing on symptoms of disease rather than diseases themselves. A limited range of symptoms characterises many common diseases, so a shift to symptoms adds flexibility without a great deal of additional complexity. Specific conditions can then be tracked by examining combinations of their symptoms. Currently, identifying the causal explanation for a detected event can be a difficult process. Our situational awareness system uses frequency statistics and cosine similarity based measures, outlined in detail in a previous paper by the authors [13], to produce terms characterising the event and then retrieve relevant news and representative tweets. Our generalised symptom focus extends to disease nowcasting. Here we use dynamic regression to fit observed symptom levels in social media to actual clinical disease count data, using influenza-like illness, gastroenteritis, diarrhoea and vomiting as case studies. We attempt to forecast future values of social media symptom data using another dynamic regression model, this time using current count data and expected movement of symptomatic individuals as the additional regressor.

The novel contributions in this paper are:

- A technique to create a data driven location network derived from social media content.
- A software system with a general symptom focus combining event detection, situational awareness, disease nowcasting and forecasting.
- A generalised disease nowcasting approach which leverages counts from multiple symptoms.
- Forecasting future symptom count levels using observed people movement from social media.

## Related Work

The term syndromic surveillance refers to methods relying on detection of clinical case features that are discernible before confirmed diagnoses are made. In particular, prior to the laboratory confirmation of an infectious disease, ill persons may exhibit behavioural patterns, symptoms, signs, or laboratory findings that can be tracked through a variety of data sources [14].

The development of Google Flu Trends as a method of harnessing Internet-scale data for syndromic surveillance [15] has led to an increasing focus on the Internet and social media as a means to obtain early warning of disease outbreaks. In that research a logistic regression was performed on the web search terms that best matched data from the US Centers For Disease Control (CDC) Influenza Like Illness (ILI) data. The correlations between the regression fit and actual CDC ILI figures were high, with mean correlations over 0.9 for all cases. However the results have since been found to be less promising than reported. According to [16] the early version of Google Flu Trends was “part flu detector, part winter detector”. Subsequent studies have addressed many of these issues, both by improved term selection using the ElasticNet and LASSO algorithms, and through dynamic recalculation of the models to fit observed ILI data [17][18][19][20].

Lampos *et al*. [4, 5] and Culotta [2] use similar methods to the Flu Trends work but apply them to Twitter data. Both studies use keyword matching to find tweets that contain flu related terms, and both find high correlations with ground truth clinical data. Other similar studies include [21] and [22]. To improve accuracy machine learning classifiers have also been developed to identify health related tweets, as in [2] and [3].

As well as monitoring the level of disease DEFENDER aims to detect outbreak events. When looking at event detection using Twitter various approaches have been attempted. These have included searching for spatial clusters in tweets [23][24], leveraging the social network structure [25], analysing the patterns of communication activity [26] and identifying significant keywords by their spatial signature [27].

Existing disease outbreak detection algorithms have also been applied to Twitter data, for example in a case study [8] of a non-seasonal disease outbreak of Enterohemorrhagic Escherichia coli (EHEC) in Germany. They searched for tweets from Germany matching the keyword “EHEC”, and used the daily tweet counts as input to their epidemic detection algorithms. Using this methodology an alert for the EHEC outbreak was triggered before standard alerting procedures would have detected it. Our study uses a modified and generalised version of this event detection approach.

When working with social media data it is difficult to apply standard epidemiological models to predict the spread and impact of disease. since parameters such as the exact type of disease and its characteristics are absent. One study [10] used the geo-located nature of tweets about illness and the fact that the social network is known to help determine the impact. They found that Twitter users who were friends of those exhibiting illness and those who had tweeted at the same time and place were more likely to subsequently become ill than randomly selected users.

In a further paper [28] the authors built on this work to develop a model allowing them to predict the future level of influenza in US cities by modelling travel patterns of Twitter users, determining that the most important factor in predicting the prevalence of flu in a given city was the number of symptomatic individuals that had flown into the city over the previous seven days.

Several software systems which detect events from Twitter and provide visualisation and situational awareness capabilities have been created in recent years. TwitInfo [29] identifies events by finding spikes in the number of tweets mentioning keywords and provides timelines and maps for visualisation. LeadLine [30] provides simliar visualisation capabilities while incorporating topic modelling and named entity recognition. Systems focused on disease include [31], [32] and [33].

## Data

Data for our work was obtained from Twitter’s live streaming API using a geographical bounding box containing England and Wales. News data was collected from 14 national and regional news sources, using a daily RSS download. For evaluation purposes we used clinical data from Public Health England in the form of the GP In Hours Weekly Bulletin [34]. The data collection period was from February to August 2014. A total of 84,438,013 tweets and 12,130 news articles were collected during this period. Retrieving tweets in this manner only returns those which contain exact geo-location information, which form around 1.6% of the total number of tweets [35].

### Symptom Focus

The system was developed with a focus on symptoms of illness. This approach was adopted since a limited range of symptoms characterise many common diseases, and the identification of disease from the symptoms presenting is itself a complex issue.

The Freebase online database [36] was used to capture a representative set of symptoms. The exact process is described in [13]. The end product was a list of 46 symptoms, each represented by groups of keywords. The number of tweets matching symptom keywords was tracked on a daily basis for each geographical area being monitored.

### Noise Removal

The primary problem with using unstructured social media data is that it is very noisy. Tweet content matching a keyword may not relate to illness at all, but be an unrelated use of the word or general discussion of illness rather than reporting (see Table 1 for example tweets).

**Table 1:** Key-term matching tweet examples.

“yet again an instance of fear over haemorrhaging of labour votes. ukip ukip.”
“my 2014 has so far been sponsored by the common cold #spons”
“sore head, sore eyes, sore legs and a runny nose. looking gooooood!”
“lets not make rash statements!”
“this game boils my blood !!!! #flappybirdsshoulddie”
“i hate hay fever -_-”

These tweets were sampled from the health classifier test set.

In order to overcome the noise issue we developed two classifiers to identify health-related content. The first is a semi-supervised Support Vector Machine (SVM) implementation that classifies tweets as being health related or not. The second is a Naive Bayes classifier that performs the same task for news articles.

The approach that has been used for tweet classification is an adapted version of the model used by Sadilek *et al*. [28]. This classifier model uses a cascading approach where an initial set of manually labelled tweets is expanded twice to form more training data for subsequent supervised classifier to use. The *LibShortText* [37] SVM library was used to perform documentation classification.

The training set of manually labelled tweets, 4600 in total, was sampled from the tweet datastore that consisted of over 30 million tweets at the time of collection. We performed a structured sample, limiting the number of each symptom group sampled from the population set. This allowed important but infrequent terms like tonsillitis or chest pain to have more instances in the sample set and boosted classification accuracy. The final classifier achieved a classification accuracy of 96.1% on a held out test set of 920 manually classified tweets.

We also developed a classifier to determine which news articles were health related. This utilises a more traditional supervised approach where training data is created by segmenting articles definitely from health only sources and those from news sources that are not exclusively health feeds. This training data was then fed into a standard Naive Bayes text classification algorithm using unigrams as features. This classifier was found to have an accuracy of 84% on a a test set of manually classified articles.

### Limitations

Our approach relies on extracting a useful signal from the Twitter data. Twitter is used by a small percentage of the population—Figures from a recent US study [38] indicate that 20% of adults use Twitter, and only some of these will post symptom information. As we only examine tweets with geo-location information, which make up around 1.6% of the total, this further limits the pool of users being sampled. This means that the signal being derived from Twitter rests on a sample from only a small fraction of the population. If that sample were random then this would not be an issue. However the US study indicates that Twitter users tend to be younger than the general population, and those who geo-locate their tweets may also be unrepresentative. Although these are genuine concerns, they also apply to many other currently used methods of syndromic surveillance. People who do not visit doctors or respond to surveys can be invisible to traditional methods, and Google Flu Trends only observes those who search for specific terms. A diversity of methods is required to capture all segments of the population. As long as the demographic bias of the Twitter data is understood from studies such as the Pew report cited above, information derived from it can be useful in a clinical context.

Our focus on symptoms rather than diseases makes it more difficult to extrapolate results into concrete predictions. Each symptom might be an indication of several different diseases, and it is knowledge of the specific disease that allows many epidemiological models to be employed. It would ideally be preferable to pinpoint specific diseases, but the issue is that these are mentioned far less frequently in Twitter discourse than symptoms. We will be investigating extrapolation of specific diseases from symptoms in further work.

## Methodology

### Location

In a previous paper [13] we developed a methodology for identifying areas of high tweet activity within a country or region. This was done by clustering the geo-located tweets using the DBSCAN algorithm. When applied to the UK the resulting areas were found to correlate with the major towns and cities.

We extend this work by considering each area identified as a node within a complete undirected graph network. This allows us to disregard the spatial size and location of the areas, forming relationships between nodes based on properties of the tweets contained within them.

**Fig 1:**
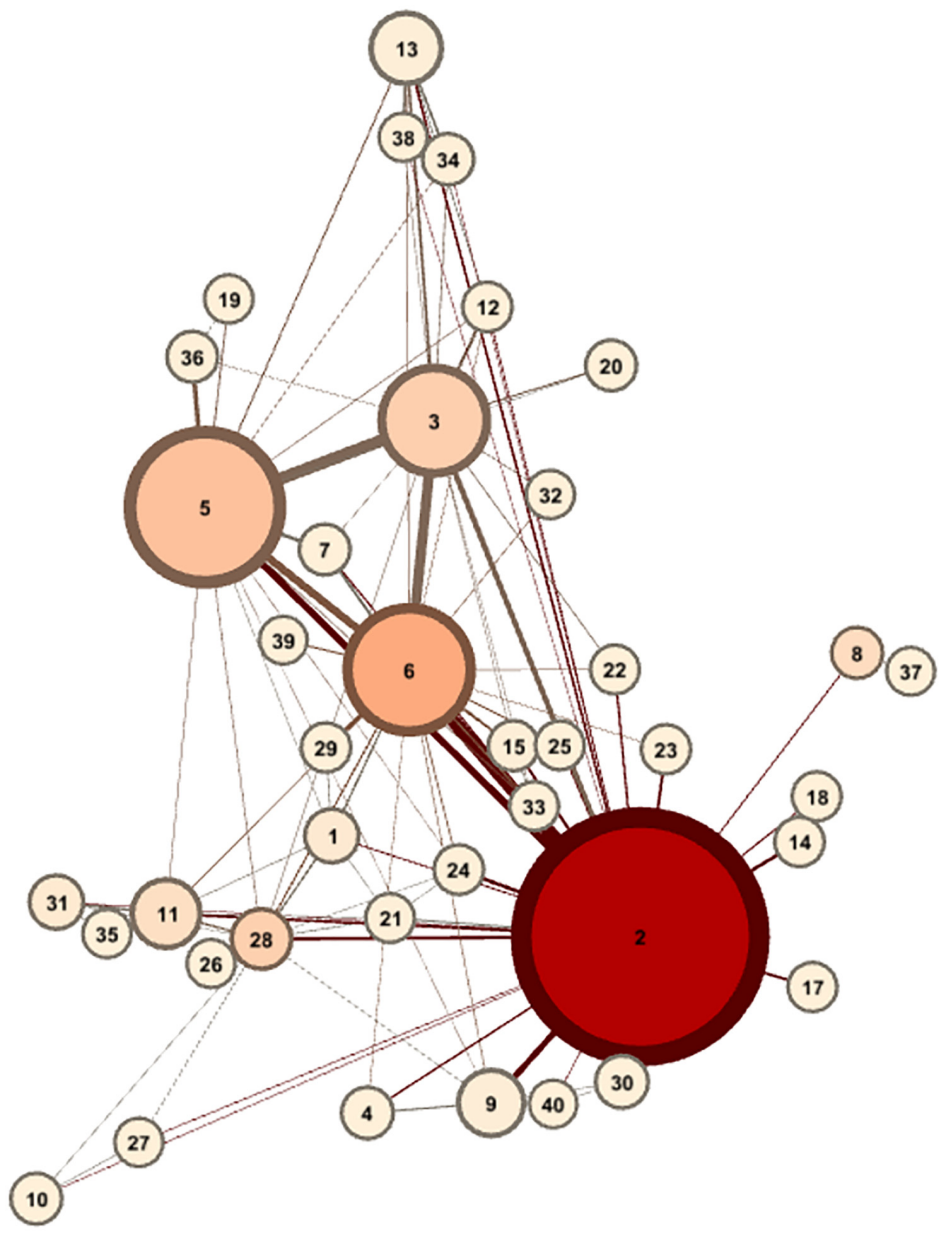
The DEFENDER Location Network for England and Wales. Nodes are coloured according to their calculated PageRank within the network. Their size corresponds to the relative average number of tweets sent from within those nodes per day. The thickness of lines between nodes corresponds to the edge weighting assigned to them. The numbers relate to the internal numbering system used for DEFENDER; The ‘London’ node is number 2 in our schema, while the ‘Birmingham’ node is 6.

The important step in construction of this network is the calculation of the weights for each edge. Since the movement of people is strongly indicative of disease transmission we use movement information derived from all of the tweets. Where a user has tweeted from more than one location in a given day this is taken as evidence that they have moved between the locations during that day and tweeted from each one. We ran queries over our data for the period of the study to find all such instances of tweeting from multiple locations on a calendar day. Each movement instance was used to increment the weighting of the edges between all nodes the user tweeted from on that day. The network generated for England and Wales with edge weights derived from this process is illustrated in Fig 1 (Only the most significant edges are shown for clarity.)

**Fig 2:**
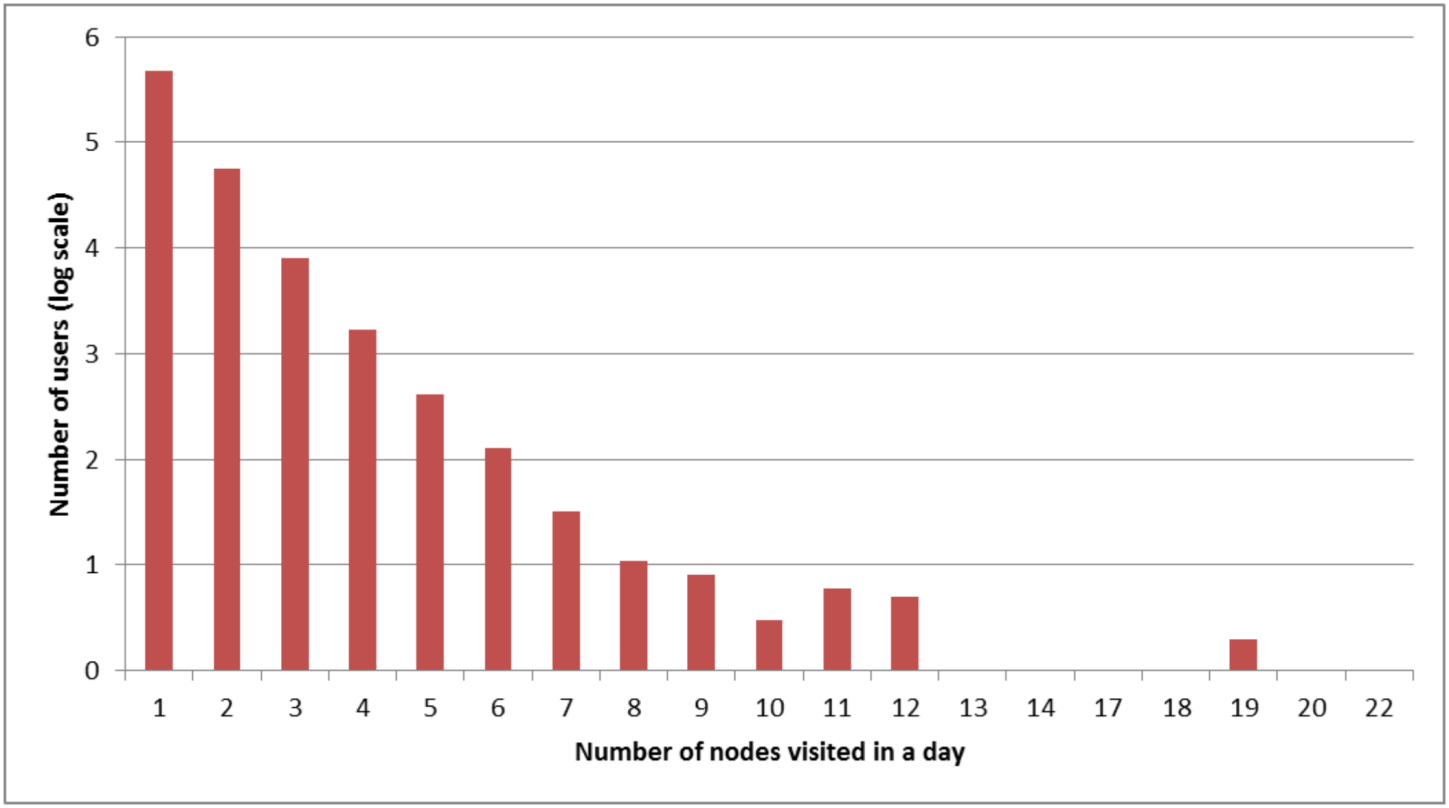
Movement characteristics of Twitter users. The number of users in our data who have moved between each number of nodes in a day.

In order to further analyse the movement data collected we took the data for the month of April 2014 as a sample. In this period 470,712 distinct users tweeted from only one place during the month, while 56,807 tweeted from 2 nodes in at least one day and 10,236 tweeted from more than 2 locations. The number of users tweeting from each number of locations is shown in Fig 2, using a log scale. 12.5% of the users in this sample therefore exhibited some movement. To examine whether those who exhibited movement differed systematically from those who did not we retrieved user profile information for all of the users concerned. This included the number of followers, tweets sent and friends (accounts they follow) for each user. The median values for these are shown in Table 2. The median was employed because the distribution of values in each category was found to be long-tailed, so mean values were not very informative.

**Table 2:** Characteristics of Twitter users by movement behaviour.

Nodes Visited	Number of users	Median followers	Median friends	Median tweets
1	470712	180	276	1352
2	56807	283	343	3518
3	7952	354	389	5448.5
4	1668	447	437.5	7356
5	414	519	499	9620
6	129	513.5	524.5	10504.5
7	32	905.5	718	14607.5
8	11	832.5	952	33571
9	8	774	48.5	16874
10	3	493	1325	36842
11	6	551.5	780.5	14446.5
12	5	610	236	36842
13	1	1068	1979	36842
14	1	368	236	337263
17	1	1109	38	16792
18	1	1109	38	16792
19	2	1088.5	1008.5	26817
20	1	1109	38	16792
22	1	1109	38	16792

The characteristics of the users who tweeted from multiple locations are clearly different from those who were more static, with increasing numbers of followers, friends and tweets sent for each increase in movement activity. This would indicate that they are more active users of Twitter. To further analyse the demographics of these users we took random samples of 100 users each from those who had tweeted from 1, 2, 3-5 and over 6 locations. Each of these users was manually assessed by examining their profile pictures and Twitter biographies. They were assessed as to the type of account (personal, company or bot), gender, age and main interests. With samples of 100, sampling theory states that there is a 10% margin for error in each category due to sampling error, at a 95% confidence interval. The results are shown in Table 3.

**Table 3:** Results of sampling Twitter users with differing movement characteristics.

Types of user	Number of nodes visited				
	1	2	3 to 5	over 5	Total
**BOT**			**2**	**9**	**11**
**COMPANY**	**11**	**4**	**1**	**1**	**17**
**DELETED**	**5**	**9**	**7**	**12**	**33**
**PERSONAL**	**84**	**87**	**90**	**78**	**339**
FEMALE	35	49	46	26	156
*MIDDLE_AGED*	*2*	*3*	*3*	*4*	*12*
*UNKNOWN*	*1*	*3*	*1*	*1*	*6*
*YOUNG*	*32*	*43*	*42*	*21*	*138*
MALE	47	37	39	50	173
*MIDDLE_AGED*	*8*	*11*	*5*	*12*	*36*
*UNKNOWN*	*2*	*3*	*1*	*2*	*8*
*YOUNG*	*37*	*23*	*33*	*36*	*129*
UNKNOWN	2	1	5	2	10
**Total**	**100**	**100**	**100**	**100**	**400**

Most accounts examined were personal rather than belonging to companies. A small number of accounts were found to be bots which spoofed the geo-location information in their tweets, so did not contain true movement information. These included accounts which tweeted local weather information, jobs and local events. These were only evident in the samples of accounts tweeting from more than 3 locations in one day. The personal accounts were split roughly evenly between males and females, within the margin of error except for accounts with very frequent movement. Far more accounts were from young people (under 35) than from middle aged or older users. The only notable trend seen in the assessment of interests was that those who had travelled to more locations were more likely to be interested in football. 5/84 of the personal accounts which had not travelled were devoted to football, rising to 14/78 in those tweeting from more than 5 locations in a day. This could be an indication of the additional travel undertaken by football supporters.

All of these results indicate that those who our methodology picks up as travelling do differ in demographics from the general population, and are more active on Twitter than most Twitter users. Further research would help to further refine the demographic nature of these users as compared to the general population.

There are multiple advantages to our data-driven generalised location system. Firstly as mentioned in [13] it is transferable to any new area within which there is sufficient tweet density to create clusters. Secondly locations which are not spatial neighbours but exhibit high population movement, for example London and Birmingham, can be considered to be “closer” than they are by distance alone. This is advantageous considering that human contact is the most important factor in spreading infectious disease [39] and thus likelihood of movement between two areas increases contact exposure chance. Finally it allows for the leveraging of existing graph based algorithms and techniques devised for analysing the structural properties of graphs such PageRank [40] and betweenness centrality.

The main disadvantage of this generalised approach to location assignment is that health statistics are generally collected using a region’s own hierarchical administrative system (for example at local authority level in the UK). Evaluation of the predictions of our models against ground truth health data thus requires mapping tweet locations back to this level, which requires some effort as shown in the results section.

### Architecture

**Fig 3:**
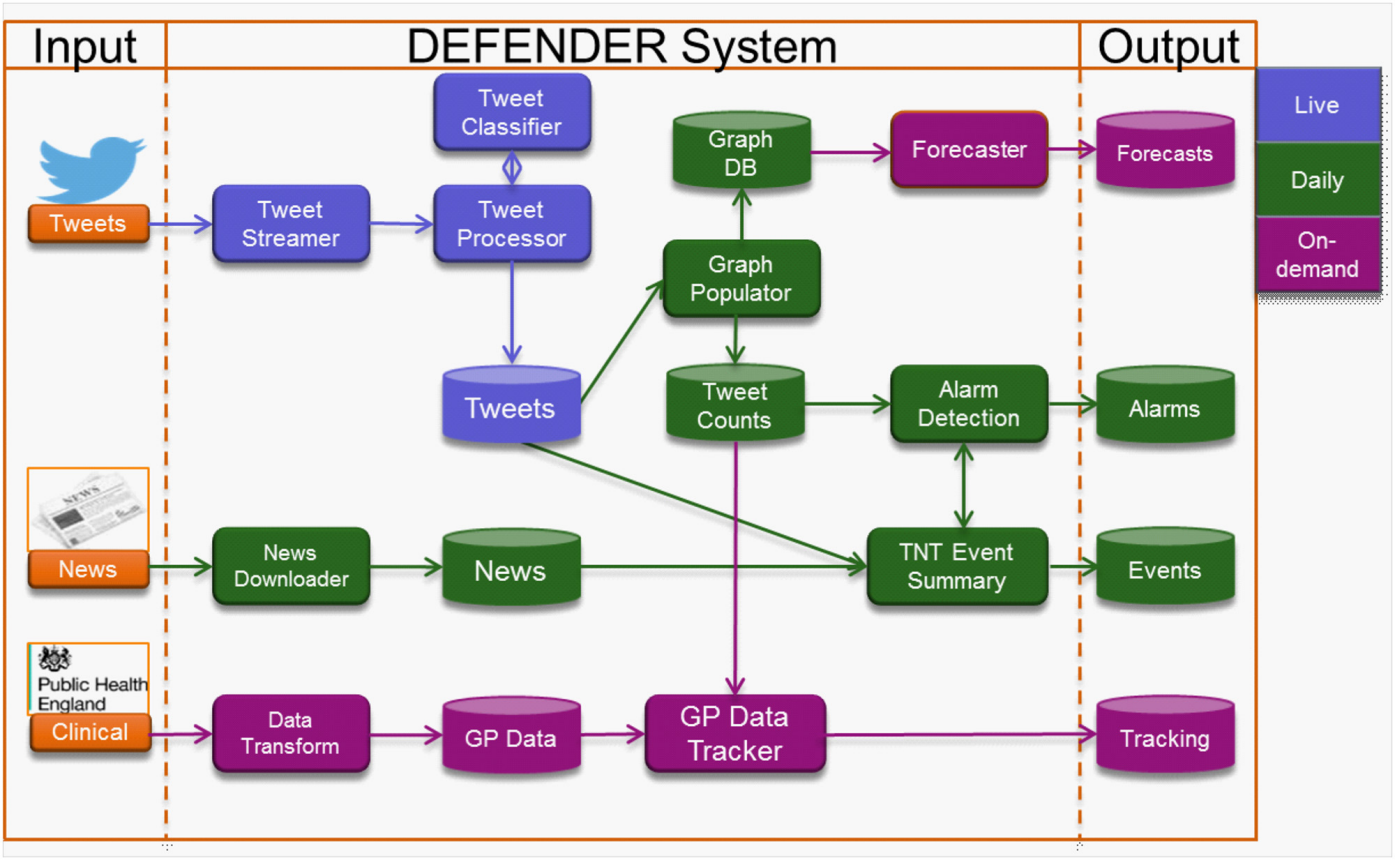
Architecture of DEFENDER. the left area shows inputs to the system: tweets, news articles and clinical data. The middle section details the internal services and data stores and the area on the right-hand side highlights the outputs which are available to be viewed or queried against. The different colours denote the data processing category.

The DEFENDER system is a working prototype consisting of a data processing pipeline backend, and a situational awareness tool using a web interface as the frontend. The DEFENDER data processing pipeline deals with data at three different temporal resolutions: live, daily and on-demand. Live is data that is collected and processed within seconds of its conception. Daily data processing tasks are scheduled to execute once a day. On-demand data is produced in response to a request from a system user. The system architecture is delineated in Fig 3.

Tweets are the primary data sources and are ingested into the system as they are posted to Twitter. The tweets are classified to ensure they are health related. Additional pre-processing then occurs where tweets are assigned to nodes in the network; named entities and symptom keyword matches are also extracted at this point. The graph populator service runs daily against the dataset of classified processed tweets. It extracts the symptom time series counts and creates a graph where the nodes (locations) have daily symptom count properties and the edges are the observed people movement between node pairs.

The graph database is used as an input by the forecasting service. The observed people movement between nodes and the amount of people exhibiting symptoms within an area provide enough information to estimate the number of symptomatic people moving into an area from its neighbours. The node/symptom tweet counts are used as input for both outbreak detection and the clinical disease nowcasting application. The Terms, News and Tweets (TNT) event summarisation service (see [13]) uses the alarms generated by the outbreak detection algorithm and fetches the source tweets from the processed tweet store with the aim of revealing any potential cause of the symptom event. The news data, which is ingested daily into the system, is also used as input to the event summarisation service. News data is retrieved from a store of news articles using search terms derived from the source tweets. The linked events (tweets and news) are stored in an event database which can be viewed via a front-end or queried directly. The disease nowcasting application attempts to forecast current actual levels of illness from lagged (one week) clinical source data. It uses GP case data and current tweet count data as inputs to its models.

### Services

#### Early Warning Detection

Early warning of disease outbreaks permits health officials to develop timely intervention strategies and can prevent large scale crises. The DEFENDER system uses an Early Warning Detection (EWD) methodology which was described in a previous work by the authors [13]. This uses the EARS syndromic surveillance algorithm to trigger alarms based on spikes in symptomatic tweet activity. By applying customised filtering criteria, including removing those alarms with a low deviation from the time series median, we are able to produce robust high confidence alarms, as evaluated in [13].

#### Situational Awareness

**Fig 4:**
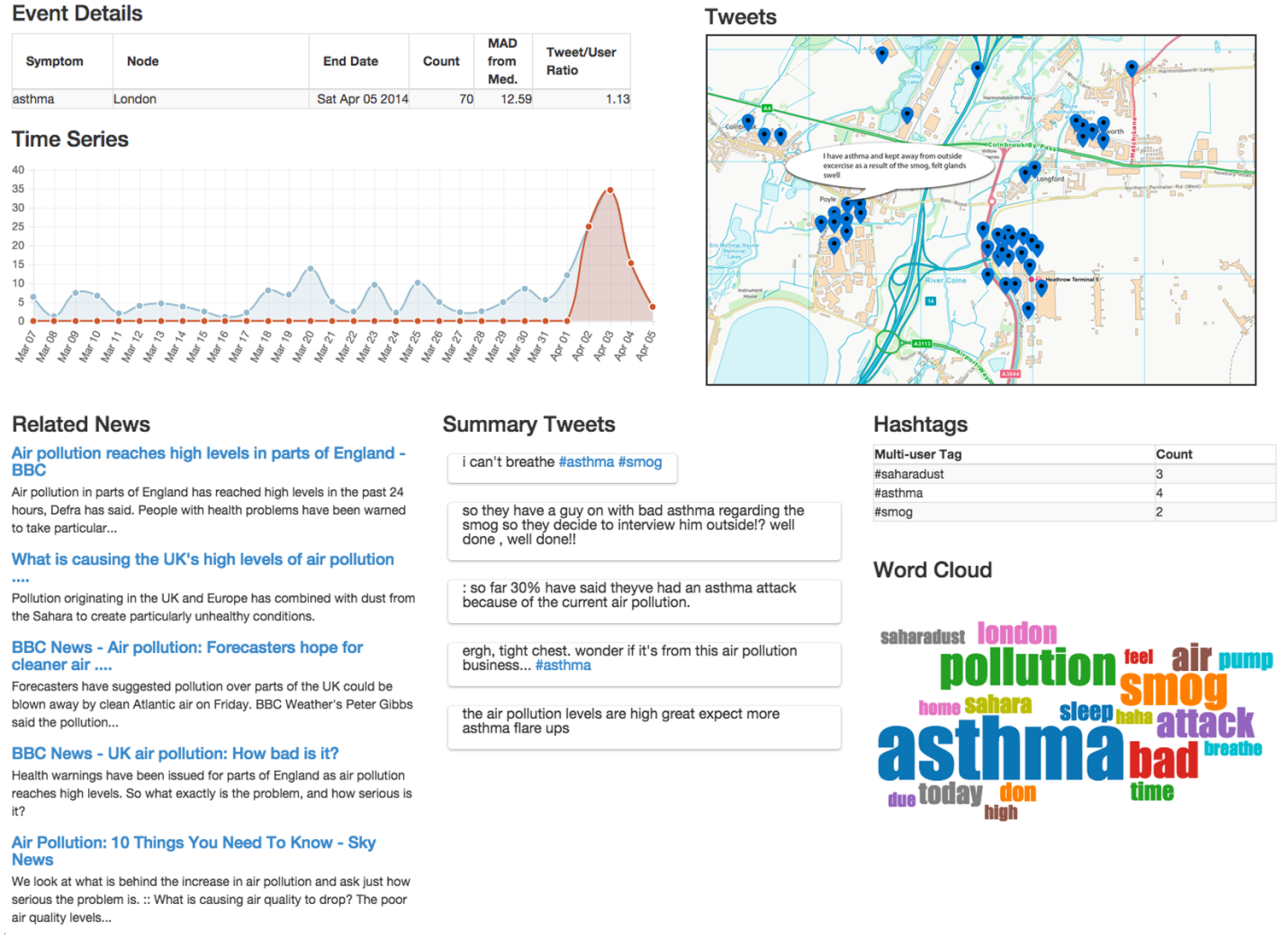
DEFENDER Situational Awareness screen showing asthma event in London from April 2014. Moving counter-clockwise from the top left the screen shows: **1. Event Details:** Basic information about the timing and severity of the event. **2. Time Series:** A graph showing the symptom counts for the previous 30 days with the alarm period highlighted in red. **3. Related News:** Most relevant news articles selected by TNT algorithm. **4. Summary Tweets:** Most relevant tweets selected by TNT. **5. Word Cloud:** Visual representation of words used most frequently in the tweets. **6. Hashtags:** Tags used by multiple users within the event. **7. Tweets:** Map showing locations of individual tweets. The map shown here is for illustrative purposes only due to copyright. Contains OS data, Crown copyright [and database right] (2016).

The situational awareness module provides the frontend of the DEFENDER application. It allows viewing the list of detected events, and gives additional context to each reported health outbreak alarm. This could be used by a public health official to determine the cause, location and importance of the alarm. We use the tweets associated with an alarm (those that match the alarm keyword and originate from the same location and time) in order to generate the situational awareness report. We use the TNT algorithm, developed for this system and described in [13], to extract terms which are specific to the event rather than the baseline of symptomatic tweets from the area. These terms potentially describe the event and can be used to retrieve relevant news articles and rank the tweets which best summarise the event. Additionally we also extract hashtags used by more than one user, frequent terms and geo-coordinates from the alarm tweet set. This event metadata is stored as part of the event along with the tweets from which they were extracted. When relevant news is found the article metadata and text is also stored as part of the linked health event. A system user can then query the event database for symptomatic events or use a front-end to provide a visual summary of a specific event. We have developed a front-end using open source web technologies as an example visualisation platform. An illustrative example of a detected event is presented in Fig 4.

#### Nowcasting

The nowcasting module aims to predict current case count data from two time series. The first is historical case data available until the previous time point, the second is tweet count data available up to and including the current time point. GP case data was retrieved for four different syndromes: Influenza-like Illness (ILI), Gastroenteritis, Diarrhoea and Vomiting at the UK local authority level from Public Health England [34]. The assumption is that adding the current tweet count data series will aid in predicting the current case count data.

The forecasting technique employed by this algorithm is dynamic regression, specifically regression with ARIMA (Autoregressive Integrated Moving Average) errors. This combines the time series focused dynamics of ARIMA models with the potential for additional regressors to explain a certain amount of variance in the data.

There are two modes of operation for the nowcasting module: 1) model training (including feature selection) and 2) symptom nowcasting using trained models. The model training algorithm uses a form of best-subset selection for choosing regression terms. A candidate set of symptom features (terms) is provided as input for the case data in question. For example flu cases may use: sore throat, fever, cough, flu, headache or other appropriate terms. From these terms all possible combinations of these terms (up to 4 terms at once) are used. The best performing keyword combinations, measured by lowest Mean Absolute Error (MAE) on a cross-validation window of 4 time periods (28 days training 7 days test) are persisted as the best model for that symptom/node combination. LASSO was also considered for term selection, but was found to be inferior to best-subset selection in this case. This might be because LASSO is most effective where the number of variables is much greater than the number of training examples, which is not the case for our data.

An interesting note is that originally the tracker used the Akaike Information Criterion (AIC) to determine the best forecast fit to the trained data and select forecast models based on this. However this was found to overfit the data and have weak predictive power for unseen data. Choosing the best fits from the MAE of 4-fold cross-validation on training data improved the accuracy on unseen data considerably. The use of such absolute error metrics has been encouraged over that of squared metrics in order to enhance interpretability, and we compare our models to naive ones in order to give a scaled error [41][42].

This is a lengthy process as it involves fitting models for all possible combinations of choosing 1,2,3 or 4 terms from all candidate terms. For example, for ILI cases in the evaluation 14 flu terms are used; the total number of model fits is then: ^14^ *C*_1_ +^14^ *C*_2_ +^14^ *C*_3_ +^14^ *C*_4_ = 1470. This however need only be done periodically. Once the best model for a GP case/location pair has been ascertained that feature set can be used to track new data in near real-time.

The nowcasting evaluation section details the results of an experiment to determine the effectiveness of this approach and compares the chosen model regression with ARIMA errors (case series and tweet count series) to alternatives models including: regular ARIMA time series forecasting, a random walk forward and a naive forecast that projects forward the mean of the existing series.

#### Forecasting

The forecasting system aims to predict the future level of tweet symptom activity across the node network. Since the system is symptom based it was not possible to employ standard disease modelling techniques such as SIR modelling, since the details of the disease such as the transmission and recovery rates are unknown. The information available to the system is a time series of the tweet count activity for each node and symptom, and also the information about the movement of people between nodes.

The assumption that was therefore made when trying to forecast future tweet counts was that knowing the future influx of symptomatic people from neighbouring regions to an area would lead to a more accurate prediction of future tweet count values. In order to estimate the influx of symptomatic people from node A to node B the following simple equation was used:

$$\begin{array}{c} \text{Influx}=\frac{\text{Symptomatic}\;\text{individuals}\;\text{in}\;\text{Node}\;\text{A}}{\text{Total}\;\text{number}\;\text{of}\;\text{individuals}\;\text{in}\;\text{Node}\;\text{A}}\times \text{Number}\;\text{travelling}\;\text{from}\;\text{A}\;\text{to}\;\text{B} \end{array}$$ (1)

In order to use this value as a regressor in a predictive model an iterative approach was used.

Assume that the system is making a forecast of tweet counts, starting at day *t*_0_. We have information on the tweet counts and influx of symptomatic people from the previous days. The model is therefore trained to use the current day’s tweet count figure and the previous day’s influx figure. A prediction is then made for *t*_1_, allowing the influx figure for *t*_1_ to be calculated. This influx figure is then used in the prediction for *t*_2_ and so on.

Several forecasting methods were trialled before an ARIMA model was settled on. A Holtz-Winters exponential moving average model was ineffective due to the lack of strong seasonality in the tweet data. Potentially this model could be applicable if several years of data were available, since many illnesses, in particular influenza, exhibit an annual pattern. A machine learning forecasting approach available in the *Weka* toolkit [43] was also trialled, but it was unable to deal with the strongly zero-weighted time series found for many node/symptom combinations, tending to predict astronomically high values if any cases appeared in the forecasting period.

The best performing models were found to be two commonly used forecasting algorithms. The first is an ARIMA model that uses the historical tweet counts alone to predict future values. This is used as a baseline comparator to determine whether the addition of movement data improves forecasting accuracy. The second adds an additional regressor in the form of future incoming symptomatic people to a node. It is hypothesised that this extra signal should confer more information than knowing the current symptom tweet activity does alone.

## Results

The DEFENDER system evaluation has been carried out by evaluating the core components: early warning detection, situational awareness, disease nowcasting and forecasting. The first two of these have been primarily evaluated in our earlier work [13]. The disease nowcasting and future symptom count forecasting algorithms are evaluated in the subsequent sections; both are evaluated by comparison to similar models. Additionally we carried out an initial analysis of our graph location network based on Twitter data.

### Location Network Analysis

When studying disease outbreaks it is important to identify movement patterns, since these can influence disease spread as well as the public health reaction. In order to examine the importance of each node in the network with respect to movement we calculated its edge-weighted PageRank [40]. PageRank is an algorithm developed for ranking web pages. An intuitive description of PageRank is that it models a random surfer clicking links on the web, with the PageRank assigned to a page being the probability of the surfer encountering it. In our model PageRank treats the movement of people within the UK as a random walk in order to determine how frequently nodes will be visited. We then ranked the nodes by PageRank and by the number of tweets assigned to them in a one month period. The top 5 nodes by each metric are shown in Tables 4 and 5 (note that these nodes do not correspond exactly to the urban areas for which they are named).

**Table 4:** Top 5 nodes sorted by Tweet Count.

Node	ID	Tweet Count
London	2	2,625,273
Manchester	5	1,422,641
Birmingham	6	1,056,275
Leeds	3	810,153
Newcastle	13	336,429

**Table 5:** Top 5 nodes sorted by PageRank.

Node	ID	PageRank
London	2	0.16370
Birmingham	6	0.10796
Manchester	5	0.08667
Leeds	3	0.07985
Bristol	28	0.03549

Examination of both lists reveals that some nodes are more central to the network than their population would imply. For example Bristol is ranked 8th by population, but moves up to 5th place by PageRank, ahead of Cardiff and Newcastle. This may be due to its location within the UK’s transport network, in which it acts as a broker between the South West, Wales and the rest of the country. In general it can be seen that central towns and those along the ‘spine’ of the UK running from Birmingham up to Manchester score more highly on PageRank, while peripheral towns score less highly. This information could be incorporated into the early warning system by upgrading the importance of alerts in those areas with a high PageRank, since individuals, and therefore diseases, are more likely to travel to and from these areas.

### Nowcasting Evaluation

The disease nowcasting evaluation covered a 132 day period from February 11th to the 22nd of June 2014. GP case data was retrieved for four different cases: Influenza-like Illness (ILI), Gastroenteritis, Diarrhoea and Vomiting at the UK local authority level from Public Health England [34].

A data mapping was performed from tweet counts in our node network to the local authority level. The local authority boundary areas were retrieved from the Ordnance Survey [44]. These were converted from a shapefile format to GeoJSON. All of the symptom cases for the period were then assigned from the local authority level to the nodes in the location system. The local authority data was provided at a weekly frequency, therefore to give daily counts so that they could be on the same scale as the tweet data, linear interpolation was used producing 7 day values from one weekly figure.

Seven different forecasting models were implemented for evaluation:

- ARIMA: autoregressive time series forecasting uses the historical case data only to predict future values.
- ARIMA with regression: uses the historical case data and tweet count information to predict future values.
- ARIMA with regression of simple moving average: uses historical case data and a simple moving average (one week smoothed value) of the tweet count.
- ARIMA with regression and weekly lagged difference between time series and regressor: performed due to the success of Lazer’s model [16], this model uses historical case data, current tweet count data and a lagged (one week ago) difference between the two values.
- Naive Control Models:
  - Mean: simple forecast projecting forward the mean of the series.
  - RWF: random walk forward: the last value in the observation series is used for all forecasted values.
  - RWF with drift: same as previous except a directional drift is included.

**Fig 5:**
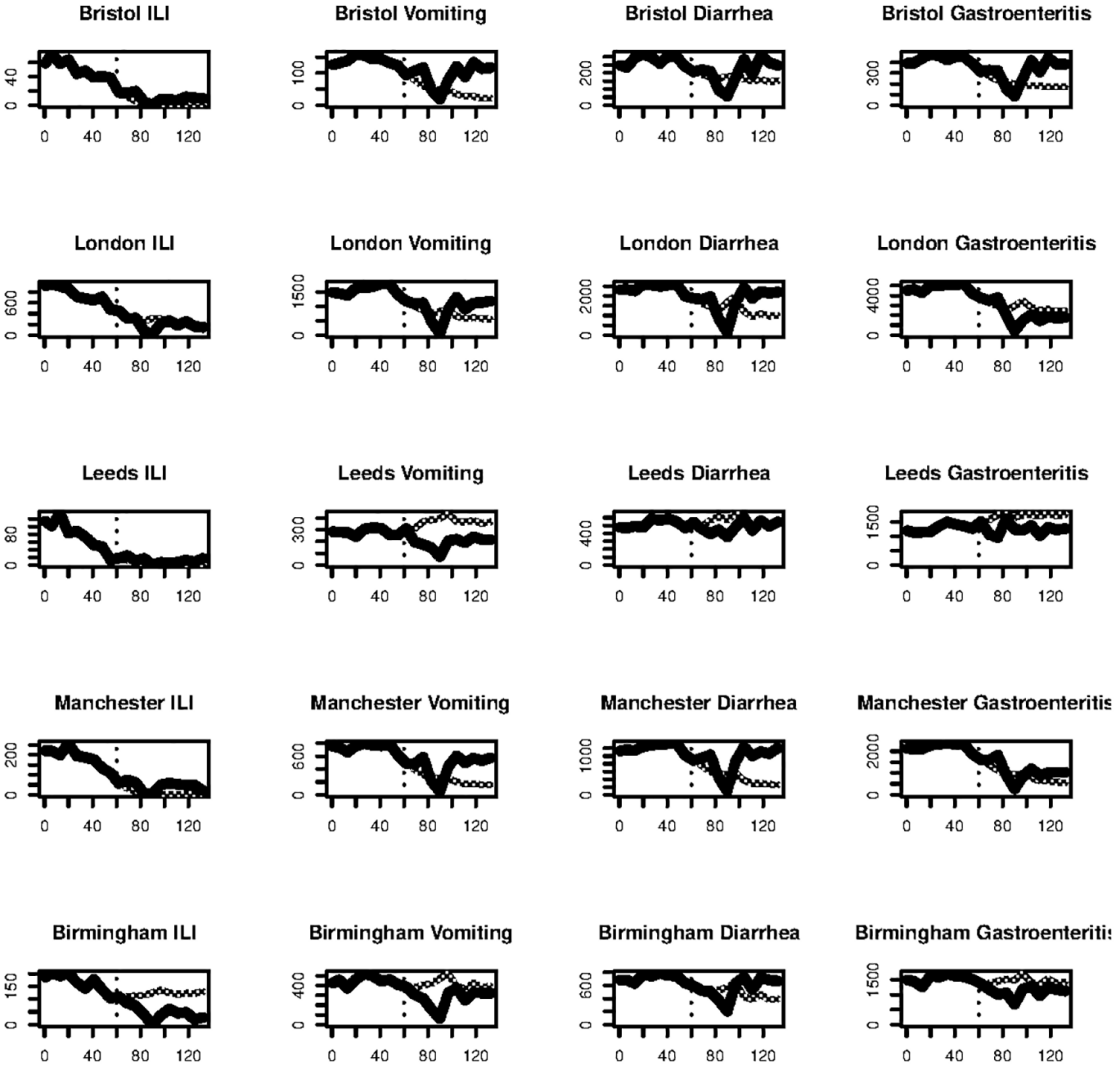
Example model outputs for 5 nodes with highest population. Each figure shows the GP case figures, with the model output after a 60 day training period indicated by the vertical line. The model shown is the ARIMA regression.

A cross-validation testing procedure was employed rather than a single train/test period, in case the position in the series affected the results. Four folds were used, each consisting of 33 days of observations (26 days training data, 7 days test). The evaluation metric used was the Mean Absolute Error, which quantifies the difference between the fitted and actual figures. This was recorded for each node/case pair. Some example model outputs are shown in Fig 5. The overall results, presented in Table 6, show that the model with combined time series forecasting and tweet count regression (ARIMA Reg.) was the best performer. This is in-line with the assumption that had been made that adding tweet count data would aid in forecasting the GP case data effectively. The regression with ARIMA errors model performed almost 40% better on average than the other models. Interestingly a similar model which performed well in [16] was beaten by the time series forecast. There are many possible choices for choosing a lagged predictor so it is left as future work to examine if any of these could improve upon these results.

**Table 6:** Mean of all MAE errors for each node/case pair.

Model	Mean MAE	Percent Diff. (Min)
ARIMA	13.05	37.21
ARIMA Reg.	8.20	0.00
ARIMA SMA Reg.	17.24	52.45
ARIMA Lag. Reg.	15.06	45.59
Mean	31.50	73.98
RWF	19.06	56.99
RWF Drift.	22.02	62.78

The percentage difference from the minimum observed value is also presented (4-fold cross-validation).

The mean of all node/case MAEs is a mean figure hence it is vulnerable to outliers distorting the results. An alternative approach to confirm the success of this model is to see the fraction of node/case pairs where the models have had the lowest (i.e. best) MAE. From Table 7 it is clear that this model is the best performer with a significantly higher fraction than all other models for all cases. These aggregate level results are from all node/case pairs.

**Table 7:** Fraction of node/case pairs where model has minimum MAE (4-fold cross-validation).

GP Cases	Arima	Arima Reg.	Arima SMA Reg.	Arima Lag. Reg.	Mean	Rwf	Rwf Drift.
ILI	0.05	0.57	0.14	0.10	0.05	0.10	0.00
Vomit	0.23	0.59	0.00	0.05	0.00	0.09	0.05
Diarrhoea	0.13	0.48	0.00	0.13	0.04	0.22	0.00
Gastroenteritis	0.00	0.57	0.09	0.04	0.04	0.22	0.04
Average (Mean)	0.10	0.55	0.06	0.08	0.03	0.16	0.02

### Forecasting Evaluation

Evaluation of the forecasting module covered the period from February 11th to June 3rd. The system was evaluated by repeatedly performing forecasts and then checking the predictions against the actual observed tweet counts. This cross-validation approach was very similar to that used in the evaluation of the disease nowcasting module. Four folds were used, each consisting of 28 days of training data and 7 days for testing. The folds started every 28 days commencing on February 11th, so the testing week for the previous fold was allowed to overlap with the training period for the next fold.

Five of the most common symptom groups were selected for testing: Sore Throat, Tonsillitis, Common Cold, Flu, Cough. In each fold, all of the above symptoms were forecast for every node, using an ARIMA autoregressive model as a baseline, and an ARIMA model including incoming tweet count data as the favoured model.

In order to obtain an accuracy measure the Mean Absolute Error was again used. For this evaluation the difference between the final forecast figure (at *t*_7_) and the actual figure was calculated at every node and an average was taken. These errors were then again averaged over all folds in order to ensure that the results were not skewed by especially favourable or unfavourable time periods.

The results are shown in Table 8, with the influx data giving a 5.8% improvement in forecasting accuracy.

**Table 8:** Mean of all MAE errors for each symptom, showing both forecasting methods. (4-fold cross-validation.)

Symptom	ARIMA	ARIMA with influx data
Sore Throat	1.37	1.43
Tonsillitis	1.44	1.41
Common Cold	1.15	1.00
Flu	1.44	1.26
Cough	1.76	1.63
Average	1.43	1.35

## Conclusion

We have created an integrated software system for monitoring disease outbreaks and generating explanations of detected events. The system provides tools for predicting the current levels of clinical case counts and for forecasting future levels of symptomatic social media activity.

The DEFENDER architecture can be easily extended to handle new symptoms and geographical regions. Adding new symptoms requires only the addition of relevant keywords. Extension to a new region requires the capture of geo-tagged tweets from the area, re-running the location clustering to generate a node network for the area and the addition of local news sources. Another main advantage is that the system can pick up signals from diseases that have not been selected in advance, due to the focus on symptoms. As long as the new disease shares symptoms with those already being tracked it will be picked up. The integrated situational awareness module allows the system to leverage the expressive power of social content combined with news media in order to provide causal explanations for detected outbreak events. These design choices do however mean that diagnostic expertise is required to interpret symptom activity detected by the system. The use of the data driven location network also means that comparisons with clinical data from administrative regions requires data mapping and transformation.

The main contributions of this paper are fourfold. Firstly we have developed a novel technique to create a data driven location network from geo-tagged social media content. Secondly we have developed a generalised disease nowcasting approach which uses counts from multiple symptoms to predict current disease activity. Thirdly we attempt to forecast future symptom count levels by employing observed people movement from social media. Finally we have built these techniques, along with those developed in an earlier paper by the authors, into a prototype of an integrated software system to aid public health officials working in syndromic surveillance.

We have evaluated each of the components of our system. In our earlier work [13] we tested the event detection and situational awareness algorithms. For event detection we examined 33 candidate alarms detected by the system. We manually assessed these alarms to identify their causes and where possible found external verification from clinical or news data that events had occurred. We compared these with those alarms tagged as genuine events by the system, producing an F1 score of 0.9362. The situational awareness component was evaluated by determining the accuracy of the news linkage and tweet ranking algorithms. The news linkage, weighted towards precision, achieved an F0.5 score of 0.79 on our example set of candidate alarms and produced no false positives at the optimum parameter level. The top ranked tweets fully matched our human-coded event summaries in 21 out of 26 cases that we examined. Evaluation performed in this paper found that the social media data was able to improve the nowcasting of diseases by 37 percent over a model that used only previous case data. The forecasting of future symptom counts provided only a moderate gain of 5.8 percent over a model using only previous count data. An initial analysis of our location network using the PageRank algorithm revealed that nodes closer to the main ‘trunk’ of the UK running from London to Manchester gained in importance compared to their population, while seaside towns were less highly ranked due to their peripheral position in the network.

In future work we aim to develop a diagnostic model linking symptoms to specific diseases, incorporate additional signals into our situational awareness and event detection modules and expand the system to different regions of the world.
